# The Evaluation of CP-001 (a Standardized Herbal Mixture of *Houttuynia cordata*, *Rehmannia glutinosa*, *Betula platyphylla*, and *Rubus coreanus*) for Cytochrome P450-Related Herb-Drug Interactions

**DOI:** 10.1155/2013/824270

**Published:** 2013-07-07

**Authors:** Hye Hyun Yoo, Sun-A Kim, In Sook Kim, Seong-Gyu Ko

**Affiliations:** ^1^Institute of Pharmaceutical Science and Technology and College of Pharmacy, Hanyang University, Ansan, Gyeonggi-do 426-791, Republic of Korea; ^2^Laboratory of Clinical Biology and Pharmacogenomics, Department of Preventive Medicine, College of Oriental Medicine, Kyung Hee University, Seoul 130-701, Republic of Korea

## Abstract

In the present study, the effect of CP-001, a standardized herbal mixture of *Houttuynia cordata*, *Rehmannia glutinosa*, *Betula platyphylla*, and *Rubus coreanus*, on cytochrome P450 (CYP) enzyme-mediated drug metabolism was investigated *in vitro* to evaluate the potential for herb-drug interactions. CP-001 was tested at concentrations of 1, 3, 10, 30, and 100 **μ**g/mL. A CYP-specific substrate mixture was incubated with CP-001 in human liver microsomes, and the metabolites generated by each CYP-specific metabolic reaction were measured by liquid chromatography-tandem mass spectrometry. CP-001 seemed to slightly inhibit some CYP isozymes, but the IC_50_ values for all CYP isozymes were greater than 100 **μ**g/mL. Furthermore, CP-001 did not exhibit time-dependent CYP inhibitory activities, indicating that it does not act as a mechanism-based inactivator of CYP enzymes. In conclusion, the effects of CP-001 on CYP isozyme activities were negligible at the concentrations tested. Therefore, the likelihood of herbal mixture-drug interaction is considered minimal.

## 1. Introduction

Herbal medicines are increasingly being used as alternative medicines worldwide. Consequently, it is very likely that some patients will take herbal medicines in combination with prescription or conventional medications, which may lead to unexpected adverse effects caused by herb-drug interactions. There may be many mechanisms underlying various herb-drug interactions, but the representative mechanism is an alteration of the absorption, metabolism, or clearance of coadministered drugs by the herb. Many herbs can induce or inhibit drug metabolizing enzymes such as cytochrome P450 (CYP450), altering the pharmacokinetics of the coadministered drugs and subsequently reducing their pharmacological effects or causing toxicity [1, 2]. For these reasons, evaluations of herb-drug interactions associated with drug metabolizing enzymes are necessary to ensure the safety of the concomitant use of herbal medicines. 

CP-001 is a standardized herbal mixture of *Rehmannia glutinosa, Houttuynia cordata, Rubus coreanus,* and *Betula platyphylla*. *R. glutinosa* has traditionally been used to treat hemostasis, activate blood circulation, and improve kidney function in East Asian countries [2]. It has been reported that *R. glutinosa *has antiallergy effects [3] and anti-inflammatory functions [4–6]. *H. cordata *has been used to treat inflammatory diseases in traditional oriental medicine, and it has been reported to possess several pharmacological activities such as anti-inflammatory, antiviral, and anticancer effects [7–9]. *R. coreanus *is a type of raspberry, the fruits of which have traditionally been used for the treatment of asthma and allergy in Asian countries and have anti-inflammatory and antioxidative activities.

Recently, in our pharmacological assay, CP-001 exhibited a synergistic therapeutic effect on atopic dermatitis (AD). This herbal mixture is currently under development as a therapeutic agent for the treatment of AD. Therefore, a preclinical evaluation of potential herb-drug interactions was required. In this study, the effects of CP-001 on CYP450-mediated drug metabolism was evaluated to predict the CYP450-related herb-drug interactions.

## 2. Materials and Methods

### 2.1. Chemicals and Reagents

CP-001 was provided by Hanpoong Pharm & Foods Co., Ltd. (Jeonju, Rebiblic of Korea). CP-001 was standardized to contain 1.25% catalpol, 0.26% quercitrin, and 0.42% ellagic acid. Pooled human liver microsomes were purchased from BD Gentest (Woburn, MA, USA). Glucose-6-phospate, *β*-NADP^+^, glucose-6-phospate dehydrogenase, phenacetin, coumarin, diclofenac, mephenytoin, dextromethorphan, midazolam, furafylline, methoxsalen, sulfaphenazole, ticlopidine, quinidine, ketoconazole, and terfenadine were obtained from Sigma Chemical Co. (St. Louis, MO, USA). Formic acid was purchased from Merck (Darmstadt, Germany). All other solvents used were of HPLC grade and were purchased from J. T. Baker (Phillipsburg, NJ, USA). Distilled water was prepared using a MilliQ purification system (Millipore, Billerica, MA, USA).

### 2.2. CYP Inhibition Assay

The CYP inhibition assay was performed with 0.5 mg/mL human liver microsomes in a final incubation volume of 0.2 mL. The incubation mixtures consisted of various concentrations of CP-001 (1, 3, 10, 30, and 100 *μ*g/mL in methanol), an NADPH-generating system (NGS) containing 0.1 M glucose-6-phosphate, 10 mg/mL *β*-NADP^+^, and 1.0 U/mL glucose-6-phosphate dehydrogenase and a substrate mixture (Table 1) in 0.1 M potassium phosphate buffer (pH 7.4). The incubation mixture was incubated at 37°C without NGS for 5 min and then continuously incubated for 30 min with NGS in a water bath. Well-known selective CYP inhibitors were tested as positive controls (10 *μ*M furafylline for CYP1A2; 20 *μ*M methoxsalen for CYP2A6; 50 *μ*M sulfaphenazole for CYP2C9; 20 *μ*M ticlopidine for CYP2C19; 50 *μ*M quinidine for CYP2D6; 5 *μ*M ketoconazole for CYP3A4). After the incubation, the reaction was stopped by adding 400 *μ*L of 0.1% acetic acid containing 4 *μ*L of an internal standard solution (16 *μ*M terfenadine in DMSO). To test the possibility of mechanism-based inactivation, 0.5 mg/mL human liver microsomes was preincubated with various concentrations of CP-001 (1, 3, 10, 30, and 100 *μ*g/mL) in 0.1 M potassium phosphate buffer (pH 7.4) for 30 min in the presence of NGS. After the preincubation, the substrate mixture was added, and the solution was continuously incubated for 30 min. The rest of the procedure was performed as described previously. 

### 2.3. Sample Preparation

The incubation mixtures were passed through activated Sep-Pak C_18_ cartridges (96-well OASIS HLB extraction cartridge, Waters, Milford, MA, USA). The cartridges were activated with methanol (1 mL) and 0.1% acetic acid in distilled water (2 mL). After sample loading, the cartridges were washed twice with 1 mL 0.1% acetic acid in distilled water and eluted with 1 mL methanol. After the evaporation of the eluates under nitrogen gas, the residue was redissolved in 100 *μ*L of reconstitution buffer (0.1% formic acid in distilled water : acetonitrile = 85 : 15), and 5 *μ*L aliquots were subjected to liquid chromatography-tandem mass spectrometry (LC-MS/MS). 

### 2.4. LC-MS/MS Analysis

The LC-MS/MS system consisted of an Agilent 1260 series binary pump HPLC system (Agilent Technologies, Palo Alto, CA, USA) and an Agilent 6460 triple quad mass spectrometer (Agilent Technologies, Palo Alto, CA, USA) equipped with an electrospray ionization (ESI) source. A Fortis C_8_ column (2.1 mm × 100 mm, 5 *μ*m; Fortis Technologies Ltd., Cheshire, England, UK) was used for the separation. The column temperature was maintained at a constant 40°C using a thermostatically controlled column oven. The HPLC mobile phases consisted of 0.1% formic acid in distilled water (A) and 90% acetonitrile in 0.1% formic acid (B). A gradient program was used for the HPLC separation with a flow rate of 0.2 mL/min. The solvent composition was initially set at 15% B, gradually increased to 85% Bover 3 min, and maintained for 1.5 min, and then the column was reequilibrated for 3.5 min. The entire column eluent was introduced directly into the mass spectrometer. Nitrogen was used both as the nebulizing gas at 20 psi and as the drying gas at a flow rate of 10 L/min at 300°C. The mass spectrometer was operated in positive ion mode. Multiple reaction monitoring (MRM) detection was employed. The precursor-product ion pairs (Q1/Q3) used in MRM mode were presented in Table 1.

## 3. Results and Discussion

The inhibitory effects of CP-001 on CYP-specific metabolic activities were evaluated in human liver microsomes. The assay system was tested with well-known selective inhibitors of CYP isozyme (positive controls). The remaining activity of the CYP isozymes after the treatment of each selective inhibitor was as follows: 5.0% for CYP1A2 (furafylline); 8.2% for CYP2A6 (methoxsalen); 5.3% for CYP2C9 (sulfaphenazole); 12.7% for CYP2C19 (ticlopidine); 2.5% for CYP2D6 (quinidine); and 4.5% for CYP3A4 (ketoconazole). All the inhibitors selectively inhibited the corresponding CYP marker activity. When CP-001 was evaluated at concentrations of 1, 3, 10, 30, and 100 *μ*g/mL, CP-001 showed minimal inhibitory effects on all CYP isozymes tested (Table 2), with estimated IC_50_ values above 100 *μ*g/mL, although the enzyme activity of CYP2C19 was slightly inhibited in a concentration-dependent manner. When CP-001 was tested after preincubation with human liver microsomes, the extent of inhibition on several CYP isozyme activities was somewhat different, but the IC_50_ values were all above 100 *μ*g/mL (Table 3), comparable to those without pre-incubation. Therefore, CP-001 is considered to have negligible effects on CYP-mediated drug metabolism as a mechanism-base inhibitor as well as a competitive inhibitor. 

There have been several reports on the effects of the herbs contained in CP-001 on CYP catalytic activities [10–12]. Regarding *H. cordata*, decreases in the activities of CYP1A1, CYP2C11, and CYP2E1 were observed in 24 h oxidized frying oil-fed rats after feeding with a diet containing *H. cordata *[10]. However, this study was not conducted to evaluate the herb-drug interaction but rather to evaluate chemopreventive potential as those CYP enzymes could be involved in the activation of precarcinogens or other chronic diseases. *R. glutinosa* was reported to inhibit CYP1A2, CYP2C9, CYP2D6, CYP2E1, and CYP3A4 *in vitro* in a concentration-dependent manner, but significant inhibition was only observed at a relatively high concentration (1 mg/mL), which is difficult to reach in a clinical setting [11]. There was a report on the effects of *R. coreanus* on CYP3A activity, but the effect was not so considerable [12]. The effects of *B. platyphylla* on CYP-mediated drug metabolism have not yet been reported. 

 In conclusion, we have evaluated the inhibitory potential of CP-001 on human CYP enzyme activities *in vitro* as a part of preclinical ADME studies. The present results indicate that CP-001 may not interact with coadministered drugs by modulating CYP-mediated metabolism. However, the results of *in vitro *tests are not necessarily consistent with *in vivo *findings. Therefore, continuous monitoring of herb-drug interactions along with pharmacokinetics studies may be required through subsequent clinical stages.

## Figures and Tables

**Table 1:** Information on the CYP-specific substrates used and the metabolites monitored in this study.

P450 isozyme	Marker substrate	Concentration	Metabolites monitored	Q1/Q3
CYP 1A2	Phenacetin	40 *µ*M	Acetaminophen	152.1/110.1
CYP 2A6	Coumarin	2.5 *µ*M	7-OH-Coumarin	162.9/106.9
CYP 2D6	Dextromethorphan	5 *µ*M	Dextrorphan	258.3/157.1
CYP 2C9	Diclofenac	10 *µ*M	4-OH-Diclofenac	312.2/230.9
CYP 2C19	(±)-Mephenytoin	160 *µ*M	4-OH-Mephenytoin	235/150.1
CYP 3A4	Midazolam	2.5 *µ*M	1-OH-Madazolam	343.1/325.1
Internal standard			Terfenadine	472.4/436.4

**Table 2:** The effects of CP-001 on the CYP enzyme activities without preincubation.

P450 isozyme	Remaining activities (% of control)					IC50
	CP-001 (*μ*g/mL)					
	1	3	10	30	100	
CYP1A2	101.6	116.1	87.5	95.6	78.3	>100
CYP2A6	83.9	81.4	85.4	88.8	94.2	>100
CYP2D6	84.5	90.1	88.1	80.4	75.2	>100
CYP2C9	122.0	130.1	93.6	111.5	108.0	>100
CYP2C19	79.4	87.1	81.8	76.8	62.0	>100
CYP3A4	87.8	96.1	86.3	88.8	85.2	>100

**Table 3:** The effects of CP-001 on the CYP enzyme activities with preincubation.

P450 isozyme	Remaining activities (% of control)					IC50
	CP-001 (*μ*g/mL)					
	1	3	10	30	100	
CYP1A2	111.4	98.8	79.3	83.5	70.6	>100
CYP2A6	116.1	106.8	104.9	101.5	96.9	>100
CYP2D6	107.3	109.6	129.2	109.3	98.7	>100
CYP2C9	83.7	96.8	75.5	76.4	69.8	>100
CYP2C19	117.5	109.7	106.3	94.2	75.1	>100
CYP3A4	111.6	109.9	102.3	89.9	75.7	>100
